# Efficient strategy to isolate exosomes using anti-CD63 antibodies conjugated to gold nanoparticles

**DOI:** 10.1186/s13568-023-01592-1

**Published:** 2023-08-28

**Authors:** Dikshita Panwar, Deepali Shrivastava, Arvind Kumar, Lavleen Kumar Gupta, N. S. Sampath Kumar, Anjani Devi Chintagunta

**Affiliations:** 1 Vignan’s Foundation for Science, Technology and Research, Guntur -Tenali Rd, Vadlamudi, 522213 Andhra Pradesh India; 2 IgY Immunologix India Private Limited, Narsingi, Rangareddy, Hyderabad, 500089 Telangana India

**Keywords:** IgY@AuNPs, Extracellular vesicles, Exosomes, ELISA, TEM, Gold nanoparticles, Tetraspanins

## Abstract

Exosomes, a subpopulation of Extracellular vesicles (EVs), are cell-secreted vesicles found in the majority of biological fluids, including breast milk, tears, sweat, blood and, urine. The density and size of these vesicles depend on a variety of factors, including age, gender and the biological condition of the individual. Researchers are now focusing on the selective extraction of exosomes from bodily fluids due to the unique biomolecule composition of exosomes, which is critical for diagnosis, disease, and regeneration. Furthermore, current approaches for exosome isolation have limitations, necessitating the development of a simpler and more effective technique to achieve this goal. In this study, we investigated a quick and effective strategy for isolating exosomes from serum using a bench-top centrifuge. This was accomplished by raising antibodies against exosome surface tetraspanins (CD9, CD63 & CD81) in Leghorn chickens due to their phylogenetic distance from humans and cost-effectiveness for commercial use. In order to separate exosomes from a complex biological fluid, the antibodies were further coupled with gold nanoparticles (AuNPs). The findings were validated using ELISA, spectrophotometry, and transmission electron microscopy (TEM). Using this technique, exosome isolation from serum was achieved rapidly and these were captured by using anti CD63 antibodies bound to AuNPs. To summarize, exosomes were purified from serum using anti-CD63 antibodies conjugated to gold nanoparticles (IgY@AuNPs). Consequently, the approach for exosome isolation from biological fluid could be useful for clinically monitoring the biological state of the patients.

## Key Points

- Exosomes have the potential to be employed as a diagnostic biomarker for the characterisation and detection of numerous pathophysiological diseases.
- We developed a reliable gold nanoparticles-mediated approach for detecting exosomes in body fluids.
- This was accomplished by employing anti-exosomal tetraspanins antibodies targeting CD9, CD63 and CD81. Based upon the findings, IgY@AuNPs (CD63) was determined to be the most efficient and were employed to capture exosomes in this study.

## Introduction

With recent progress in the field of biomarkers research, exosomes have secured a great position, consisting the advance usage in drug discovery and therapeutics. Exosomes can be employed as medication delivery vesicles for several diseases, such as diabetic fibrosis and brain sickness. These are also used in the diagnosis and imaging of diseases like Alzheimer’s disease, Parkinson's inflammation, and cancer autoimmune metastasis (Huda et al. 2021). In addition, exosomes are frequently used as a biomarker and a tool for early detection in liquid biopsies using biological fluids, RNA, DNA, and proteins (Huda et al. 2021). Therefore, exosome-mediated detection and characterization techniques have emerged as a potential and robust tool for disease diagnosis. Exosomes are naturally occurring cell components that mediate intercellular signalling and range in size from 20 to 150 nm (Zhu et al. 2022; Zhang et al. 2019). These are distinguished by their bioactive components, which vary from cell to cell and include proteins, lipids, DNA, and RNA (Zhang et al. 2019).

Exosome biogenesis, in contrast to other extracellular vesicles, originates inside multi-vesicular bodies (a subset of late endosomes), where intraluminal vesicles (ILVs) are produced. These ILVs either fuse with the lysosome for degradation or connect with the plasma membrane to release the ILVs, also known as extracellular exosomes. (Zhang et al. 2019; Gurunathan et al. 2021). Exosomes are desirable as therapeutic vectors because of their reduced immunogenicity caused by biocompatibility and a bi-layered lipid structure that shields the genetic payload from destruction (Gurung et al. 2021). Tetraspanins family members (CD9, CD63 & CD81), endosomal sorting complex needed for transport (ESCRT) proteins (Alix, TSG101), heat shock proteins (Hsp), integrins, actin, and flotillins are the primary membrane-bound and cytosolic proteins of exosomes (Gurung et al. 2021). Tetraspanins can interact with a variety of receptors and signaling molecules at the membrane. Hence these either may be involved in the exosome’s attachment and its absorption by target cells, or antigen presentation as a response of the immune system (Gurung et al. 2021).

Exosomes, being such a valuable resource in research, it is unfortunate that exosomes isolation methods including size-exclusion chromatography, precipitation, ultrafiltration, immunoaffinity and ultracentrifuge are available with lots of limitations such as slow processing volume, time-consuming, low purity, dependency on expensive instruments (Pammi et al. 2022). However, commercial exosome isolation kits are available, such as (Invitrogen, USA) (TEI), Exo-spin (Cell guidance systems, UK) (ExoS), Eloquence (System Biosciences, USA) (ExoQ), and and so forth (Chen et al. 2022). These have the advantages of quick isolation and ease of handling, but the primary drawback is that these are expensive and not ideal for mass sample processing (Pammi et al. 2022). The present study reports a gold nanoparticle based quick and effective approach for exosome detection in human serum using anti-exosomal tetraspanins antibodies. Based on the specificity of anti-tetraspanins antibodies, immunoaffinity capture offers a superior exosomal population (Ludwig et al. 2019). Research performed in the human cell line, HEK293, analysed that CD9, CD63, and CD81 was the most highly enriched protein of exosomes and hence have been utilized for exosome capturing and detection (Fordjour et al. 2022). This distinct pattern of tetraspanins expression provides an antibody-capture and detection of a subpopulation of extracellular vesicles i.e., exosomes through AuNPs (Mizenko et al. 2021; Parthasarathy et al. 2009).

Since the Middle Ages, gold has been used in the biomedical field to aid in the treatment of various diseases such as arthritis, epilepsy, heart difficulties, venereal disorders, and the diagnosis of syphilis (Bai et al. 2020). As a result of the growing understanding enabled by modern nanotechnology, biomedical applications of AuNPs such as imaging agents, drug delivery, nano-enzymes, irradiation, targeting, and photothermal therapy have been significantly advanced (Bai et al. 2020). AuNPs have exceptional physicochemical features that enable them to make a stable chemical interaction with S- and N-containing groups, giving them an edge over other nanoparticles (Bai et al. 2020; Yeh et al. 2012). These surface modifications give AuNPs exceptional biocompatibility and the ability to bind a variety of organic ligands or polymers for various purposes (Bai et al. 2020; Yeh et al. 2012). These nanomaterial-based biosensors have been widely used in liquid biopsies for tumor-derived exosomes as a source of cancer biomarkers. Here in this study, we have utilized AuNPs synthesized at temperature variation (99 ℃ and 80 ℃) as a detecting and tracking agent for targeted antibodies raised against the exosomal tetraspanins i.e., CD9, CD63, and CD81. This is made possible by the various functionalized groups present on the surface of AuNPs, which are involved in nanoparticle stability, functionality, biocompatibility, and the development of novel properties (Jazayeri et al. 2016). Low molecular weight ligands, peptides, proteins, polysaccharides, polyunsaturated and saturated fatty acids, DNA, plasmids, and siRNA are all examples of functionalized groups that can be attached to nanoparticles (Jazayeri et al. 2016). Raised antibodies were bound to AuNPs in this study via physical interactions such as ionic interaction, hydrophobic interaction, and dative binding (Jazayeri et al. 2016). AuNPs, on the other hand, have a tendency to coagulate due to their highly reactive nature that must be stabilized for long-term storage. However, polyethylene glycol (PEG) could be used to resolve this, as its hydrophilic properties boost the biocompatibility and stability of conjugated complexes (Jazayeri et al. 2016; Reznickova et al. 2017).

In this paper, we describe a rapid and effective immunoaffinity technique based on gold nanoparticles, in which anti-exosomal antibodies have been decorated on AuNPs through physically reactive interactions. These antibody-conjugated gold nanoparticles are processed further by centrifugation after being treated with human serum. The spectrophotometry and TEM were used to analyse and characterize the work described above.

## Materials and methods

### Materials

Several chemicals have been used to carry out various experiments in the present study. Chemicals such as gold (III) chloride hydrate (HAuCl4: Cat No. 254169) and poly (ethylene glycol) 2-mercaptoethanol ether acetic acid (PEG 3500 Da, Cat No. 757837) have been purchased from Sigma Aldrich whereas trisodium citrate dihydrate (Cat No. 85919), sodium hydroxide (NaOH (Cat No. 68151), bovine serum albumin (Cat No. 83803), Coomassie Brilliant Blue G-250, Methanol extra pure AR 99.8, Orthophosphoric acid extra pure 85%, Whatman^®^ cellulose filter papers, bovine serum albumin (Cat No. 05470), Sodium citrate, citric acid, Sodium periodate, Ethylene glycol, Sodium carbonate/bicarbonate, Sodium cyanoborohydride, Glycerol, and Thiomersal have been purchased from SRL Chemicals. Non-fat milk powder has been purchased from a local vendor. Primary antibodies, anti CD9, CD63 & CD81, were raised in chickens for commercial use. Secondary antibodies were conjugated with Horseradish peroxidase through sodium periodate methods.

### Methods

**Fig. 1:**
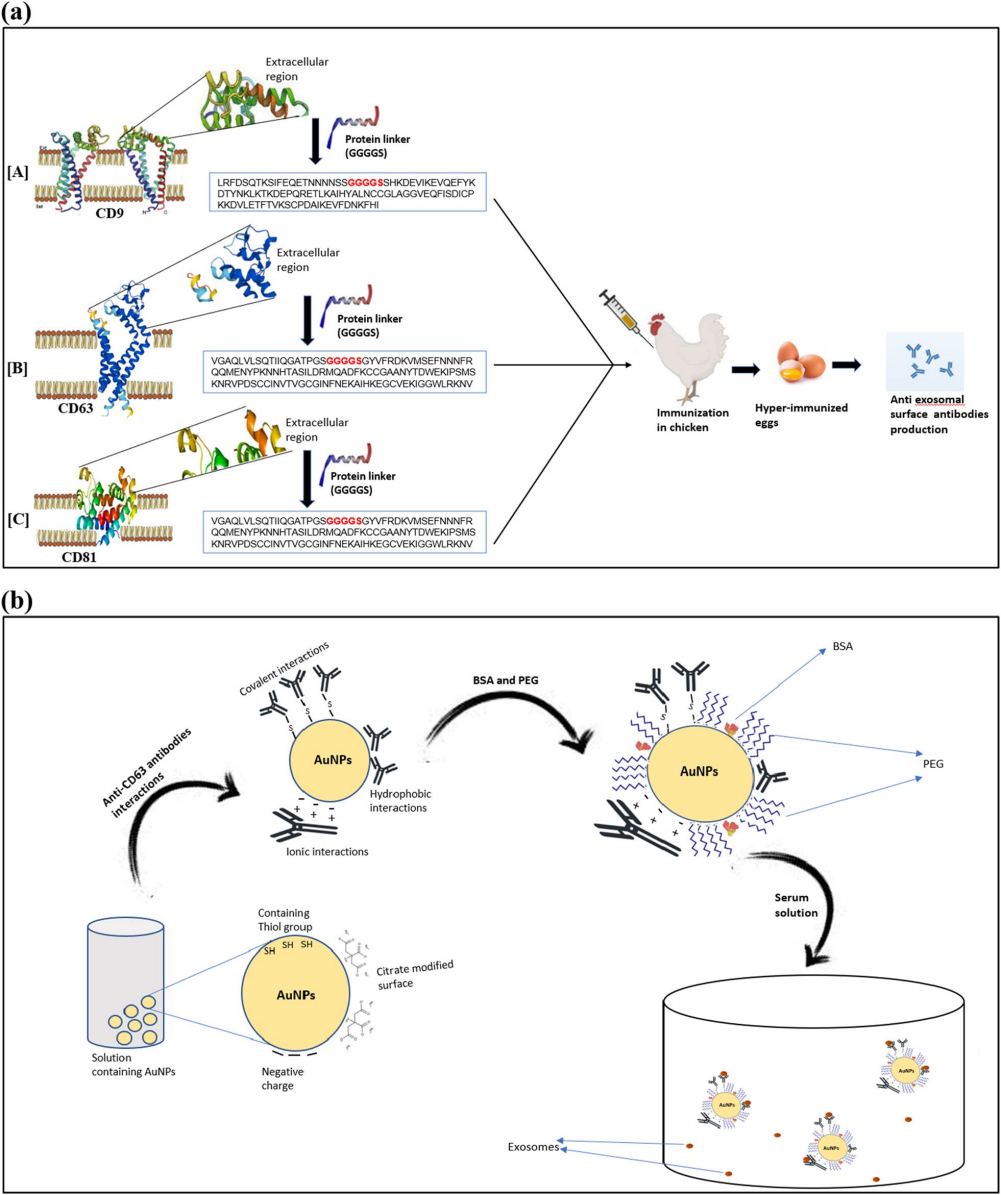
**a** A simplified workflow depicting the introduction of antigen and the production of antibodies in chicken, **b** Systematic diagram representing the synthesis of AuNPs and their conjugation with IgY antibodies for exosome capture

The overall workflow of this study is briefly described in Fig. 1. The three exosomal surface tetraspanins, CD9, CD63, and CD81, were selected to produce functionalized antibodies to capture exosomes. In order to raise antibodies in chicken, the extracellular portion of these tetraspanins was selected as an immunogen. Antibodies affinity was confirmed through Enzyme-linked immunosorbent assay (ELISA) using spectrophotometry techniques. Furthermore, freshly synthesized citrate passivated gold nanoparticles were synthesized and coupled with the anti-exosomal antibodies. This antibody-exosome coupling was investigated using an omega spectrophotometer and transmission electron microscope. The antibodies bound to AuNPs (IgY@AuNPs) were finally introduced into human serum for exosome capture, which was further determined through TEM studies.

### Characterisation of tetraspanins for antigenic epitope detection

Tetraspanins are members of a protein family with intracellular N-and C-termini, two extracellular domains (EC1 and EC2), and four transmembrane domains (Van et al. 2017).

Although there are different tetraspanin varieties in each phylum, their chemical composition which includes four or more cysteine residues in a CCG motif of the EC2 domain is remarkably consistent across species (Van et al. 2017; Huang et al. 2005). Furthermore, antibodies that target surface markers such as tetraspanins proteins (CD9, CD63, and CD81) are used in several isolation and characterization methods for various extracellular vesicles, including immunoaffinity purification, flow cytometry, ELISA, and Western blot. (Jiang et al. 2020). Moreover, the extracellular regions of these tetraspanins were targeted for antibodies production in this study, and the protein sequence was retrieved from the NCBI database. Among the various populations of EVs, the extracellular region of exosomal tetraspanins has high accessibility and is easy to target for their capture.

### Designing and synthesizing of antigen for antibody production

Tetraspanin is comprised of up of four membrane-spanning domains, with a short cytoplasmic N and C terminus. Since the extracellular region was selected as an antigen for antibody production, a linker protein sequence is required to connect the two extracellular loops of the tetraspanins. The foundation of linker design is analogous to linkers derived from naturally occurring multi-domain proteins. Researchers’ generally classified the linkers into three types based on their structures: rigid linkers, flexible linkers, and in vivo cleavable linkers. Among these, flexible linkers are generally rich in small or polar amino acids like Glycine and Serine to provide good flexibility and solubility, and they are considered more appropriate for movements and interactions required for fusion protein domains (Chen et al. 2013). Here a flexible linker sequence GGGGS has been used, due to its advantages, like ensuring correct protein orientation and not interfering with the folding of the protein domain. In addition to this, the linker sequence was also reported for providing stability of fusion protein and enhancing protein expression (Chen et al. 2013). The selected extracellular region of CD9, CD63, and CD81 tetraspanins were joined with this linker, and the resulting nucleotide sequences were optimized through integrated DNA technologies (IDT), an online available oligo optimization tool. Gene Universal, Delaware, USA, synthesised the codon-optimized gene sequence for the defined extracellular region. The desired gene was synthesized and cloned in the PET28a vector. Furthermore, the vector was transformed into BL21 Codon+ cells.

The entire amino acid sequence data for CD9 were obtained from UniProtKB (Swiss-Prot) using the reference URL https://www.uniprot.org/uniprotkb/P21926/entry and accession number: P21926. Similarly, the reference URL and accession number for CD63 were https://www.uniprot.org/uniprotkb/P08962/entry and P08962, while the reference URL and accession number for CD81 were https://www.uniprot.org/uniprotkb/P60033/entry and P60033.

### Transformation of PET28a into BL21 Codon plus competent cells

The plasmids (PET28a) with the desired genes, i.e., CD9, CD63, and CD81, were transformed through the heat shock method into BL21 codon plus competence cells, which is a strain of *E. coli.* In this investigation, competence cells were prepared using the Li et al. method for increasing cell competence efficiency (Li et al. 2010). Furthermore, the transformation procedure was customized as per the requirement with the plasmid concentration of 5 ng and an antibiotic concentration (Kanamycin) of 34 μg/ml (Li et al. 2010).

### Protein expression

The protein expression was modified based on a previous study, and the steps that follow are described briefly (Feng et al. 2006). The transformed single colony of BL21 codon plus was subjected to a Luria–Bertani (LB) medium containing 34 µg/ml of Kanamycin (Kan) for overnight growth at 37 ℃. Furthermore, 3% of this overnight culture was inoculated in 50 ml of terrific broth (TB) prepared in phosphate-buffered saline **(**PBS) supplemented with 0.5% glycerol and 30–34 µg/ml of kanamycin, followed by the incubation at 37 ℃ till its mid-log phase (0.6–0.8 OD) at 600 nm. The addition of 0.5 mM Isopropyl ß-D-1-thiogalactopyranoside (IPTG) induced protein expression and the culture was incubated for another 3 h at 37 ℃ with aeration at 250 rpm of stirring. Centrifugation at 6000 g for 10 min at 4 ℃ was used to collect the cells, and pellet wash buffer (50 mM Tris–HCl and 100 mM NaCl) was used to clean the pellet. All three tetraspanins (CD9, CD63 & CD81) extracellular proteins were expressed with the identical approach, described above.

### Protein purification from inclusion body

After bacterial cell lysis, it was noted that the desired protein attained in the form of an inclusion body (IB) which is extremely dynamic in nature, and its molecules can reversibly disaggregate and assemble back into their native state (Peternel et al. 2010). In contrast to mechanical processes like sonication or homogenization, cell disruption by chemical lysis is considered more effective in the case of an inclusion body (Peternel et al. 2010). The bacterial pellet was resuspended and homogenized in lysis buffer-1 (0.2 mg/ml lysozyme, 10 µg/ml DNase, 50 mM Tris pH 8.8) for chemical lysis and incubated overnight at 37 ℃ in a rotator at 100 rpm. Following that, lysis buffer-2 (1 M NaCl and 1% Triton X-100) was added to the same tube at a 1:1 volume ratio of lysis buffer-1 and lysis buffer-2. The inclusion bodies were collected after complete lysis by centrifugation at 8000 g for 10 min, followed by 4 to 5 washes with wash buffer (100 mM NaCl and 50 mM Tris, pH 7.5). In addition, the IB pellet was re-suspended in denaturing buffer (6 M Gu-HCl in 50 mM Tris, pH 7.4) and incubated at 4 ℃ for 30 min to denature the inclusion body. The denatured IB was centrifuged at 9000 g for 10 min, and the supernatant was collected for further protein renaturation. Furthermore, the supernatant was dropped into renature buffer (20 mM MgCl_2,_ 20 mM Trehalose, 100 mM NaCl, 5% Ethylene Glycol, 0.01% Tween80, 100 mM L-Arginine, 50 mM Tris, 1 mM GSSG, 10 mM GSH) while being continuously stirred at 4 ℃. The aforementioned mixture was subsequently incubated at 4 ℃ for 48 h, and then the refolded protein was concentrated using sucrose. Additionally, the purity of refolded proteins (CD9, CD63, & CD81) was analysed through SDS-PAGE. Considering the previous studies, the performed experiments for protein expression and purification in this study were modified based on the requirements (Palmer et al. 2012; Singh et al. 2005; Yang et al. 2011).

### Immunization of antigen into chicken

To raise the anti-exosomal antibodies in a cost-effectively, chicken has been preferred due to its phylogenetical difference between avian and mammalian species which enhances the sensitivity of immunological assays. In 8-month-old white leghorn chickens, the purified protein of three exosomal tetraspanins (CD9, CD63, & CD81) was injected intramuscularly every 14th day for a total of 8 weeks. Each bird received approximately 100 µg of protein dissolved in 150 µl of PBS and 350 µl of Montanide adjuvant (Seppic Inc., US) on the day of immunization. The bird that received only PBS and adjuvant was employed as a negative control. By stimulating the B-cell response, adjuvants accelerated the immune system's response to the antigen (Schade et al. 2005; Sunita et al. 2020). Afterwards, the eggs were collected for IgY antibody purification and their immunoaffinity was assessed using the ELISA method.

### Antibodies purification

Based on the evaluation of earlier studies for protein purification, the isolation and purification of antibodies from the immunized chicken were carried out using the egg yolk component (Sunita et al. 2020; Panwar et al. 2023). In a nutshell, egg yolk from immunized chicken eggs was diluted in deionized water at a 1:10 ratio and homogenized to make a uniform suspension. The pH of the above mixture was then adjusted to 5.0 using 0.1 N HCl before being stored at − 20 ℃ overnight. To precipitate all the lipids and phospholipids while leaving the protein in the supernatant, the frozen sample was defrosted at room temperature the following day without being shaken. NaCl at a concentration of 8.8% was then added to the supernatant after it had been filtered and collected. Furthermore, the pH of the above solution was adjusted to 4.0 with 0.1 N HCl followed by the incubation at room temperature for 1 h. The precipitated protein holding IgY antibodies was collected by centrifugation at 5000 rpm for 10 min and resuspended in the required volume of PBS. The protein purity and the concentration were determined using the SDS-PAGE and UV absorbance method respectively. The purified antibodies were stored at − 20 ℃ for further usage.

### SDS-PAGE

SDS-PAGE, as a simple tool, has been widely used for a long time to assess the purity of desired proteins. In this study, the traditional method described by Laemmli has been followed to analyse the purity of antibodies as well as refolded proteins (Muhammad et al. 2018). As a molecular weight marker, a protein ladder with a range of 20–95 kDa (SRL Chemicals), was utilized in this study. Afterward, the gel was stained with Coomassie Brilliant Blue for visualization.

### Quantitative analysis of antibodies affinity through ELISA

Antibody titration was performed through Indirect-ELISA to analyse the antibody affinity towards the antigen. Briefly, in flat bottom microtiter plates (Thermo Scientific, USA), antigen was coated (as 500 ng/well) in coating buffer (100 mM sodium carbonate, 100 mM sodium bicarbonate, pH 9.5) followed by the addition of 300 µl blocking buffer (5% skimmed milk prepared in Phosphate Buffered Saline with Tween 20). Furthermore, antibodies at various concentrations (100 µg/ml to two-fold dilution till seventh well) were prepared in diluent buffer (1/10 dilution of blocking buffer in PBS) and added to the wells, along with negative control (without antibodies) in eighth well. Anti-chicken antibodies, as a secondary antibody was diluted in diluent and introduced into the wells at a 1:6000 ratio. Finally, colour was developed by the addition of substrate buffer (100 µg/ml) of Tetramethylbenzidine (TMB), 0.01% H_2_O_2_ in 50 mM citrate buffer, pH 5.0) followed by the addition of stop solution (1N H_2_SO_4_) after 20 min incubation in dark at room temperature and optical density was measured by FLUOstar Omega spectrophotometer (BMG LABTECH’s). The ELISA volume was kept as 100 µl/well. Excluding the substrate step, a 1 h incubation at 37 ℃ was followed by four washes with PBST (PBS containing 0.05% Tween20) used in this assay.

### Gold nanoparticle synthesis

Nanoparticles were synthesized using the conventional method, by dissolving gold chloride in trisodium citrate at 85–90 ℃ and resulting in a chemical reduction of gold (Polte et al. 2010). Here the concentration of gold chloride and tri-sodium citrate was used at 0.5 mmol/l and 5 mmol/l respectively. All glassware must be treated with aqua Regia (1:3 ratio of HNO_3_ and HCl) overnight at 37 ℃, then thoroughly washed with double distilled water and dried before use. Briefly, the gold was introduced into an aqueous solution (triple distilled water) and heated while stirring in a temperature-controlled stirrer. Trisodium citrate was immediately added after it reached the desired temperature. Then it was allowed to continue stirring for another 10 min until the colour of the gold solution changes from yellowish to colourless to wine red followed by a 20 min further incubation at the same temperature. The NPs were fabricated at two distinct temperatures (99 ℃ and 80 ℃) for a better understanding of the AuNPs size variation. The sample was extracted carefully, after being cooled at room temperature, by using the pipette for UV analysis and pH measurement (approx. 5.6–6.0).

### Antibody decoration on AuNPs

The citrate-mediated AuNPs were conjugated with the antibodies through physical methods including ionic interactions, interaction with the thiol group, and hydrophobic interactions. To start with the removal of citrate and activation of AuNPs using 0.25% Tween-20, followed by incubation at room temperature for 2 h while stirring. After centrifuging the gold nanoparticle suspension at 13,500 rpm for 20 min, the supernatant is collected and resuspended in the phosphate buffer (pH 7.6). Additionally, the AuNPs were washed twice with phosphate buffer (10 mM, pH 7.2) and resuspended again in the same buffer. The washed NPs were adjusted to pH 8 using 100 mM of NaOH. Furthermore, antibodies were diluted in phosphate buffer to desired concentration (0.2 mg of all three antibodies/1 ml of AuNPs used in this) and added dropwise with continuous stirring followed by incubation of 2 h at room temperature while shaking. Then the NPs were blocked with the addition of 0.2 mg of BSA followed by centrifugation at 12,000 rpm for 15 min to remove the unbound antibodies. Afterward, the conjugated NPs were resuspended in phosphate buffer and 0.25% of PEG and 0.5% glycerol were added for stabilization. The above-described methodology was influenced by various previous studies (Juan et al. 2020; Goossens et al. 2017; Okyem et al. 2021).

### Isolation of exosomes from serum

For this purpose, blood was collected from healthy volunteers in clot activator tubes to allow the blood to clot in an upright position for 1 h at room temperature. During this period, the RBCs settled and the pure serum was collected by centrifugation at 5000 rpm for 15 min, followed by aliquoting of 1 ml serum in 1.5 ml tubes and storing at − 20 ℃. For exosome isolation, 1 ml of the twofold diluted serum was incubated with 1 ml of anti-exosomes antibodies conjugated with AuNPs for 1 h at room temperature. Furthermore, the nanoparticles have been washed completely twice and resuspended in phosphate buffer for further analysis through ELISA or TEM.

### Sample preparation for TEM analysis of AuNPs and IgY@AuNPs

The IgY@AuNPs complex bound with exosomes and plain AuNPs has been considered as a sample for TEM analysis. On a TEM grid, 10 µl of each sample was applied sequentially and the excess volume was wiped away with filter paper. The TEM grid was washed and air-dried three times before proceeding to the next sample. Each sample has been analysed twice for better understanding and clarity.

## Results

### Designing the antigen sequence

Tetraspanins, CD9, CD63, and CD81, encode proteins with two extracellular and two intracellular loops. The extracellular loops are rich in numerous disulfide linkages that are conserved across the tetraspanin family, and palmitoylation sites aid in protein and lipid interactions. Moreover, CD9, CD63, and CD81 all encode surface integral proteins that are, respectively, 228, 238, and 236 amino acids long. According to the National Centre for Biotechnology Information (NCBI), the extracellular region of CD9, which was used as an antigen, spanned the lipoprotein membrane from 34 to 55 amino acids and 112 to 195 amino acids. Similarly, the extracellular region of the other two tetraspanins, which span 33–51, 103–203 for CD63, and 34–63, 113–201 for CD81, has served the purpose. Each tetraspanin's extracellular domain was joined by the GGGGS linker to construct a protein sequence that is 112, 124, and 127 amino acids long for CD9, CD63, and CD81, respectively (Table 1). The nucleotide sequence has been revealed for the synthesis of these three proteins which was designed with the addition of restriction enzyme sites, namely *Nco*1 and *Xho*-1. The constructed sequences for the extracellular regions of CD9, CD63, and CD81 have the following accession numbers: OR166431, OR166432, and OR166433, respectively. The codon-optimized sequence (Table 2) was cloned in PET 28a plasmid by Gene Universal and expressed in a strain of *E. coli* i.e., BL-21 codon plus.

**Table 1:** Representing the selected sequence for the extracellular region of tetraspanins with their molecular weight

Name	Protein sequence	No of amino acid	Mol.wt
CD9	WLRFDSQTKSIFEQETNNNNSSGGGGSYSHKDEVIKEVQEFYKDTYNKLKTKDEPQRETLKAIHYALNCCGLAGGVEQFISDICPKKDVLETFTVKSCPDAIKEVFDNKFHI	112	12.3 kDa
CD63	VGAQLVLSQTIIQGATPGSGGGGSGYVFRDKVMSEFNNNFRQQMENYPKNNHTASILDRMQADFKCCGAANYTDWEKIPSMSKNRVPDSCCINVTVGCGINFNEKAIHKEGCVEKIGGWLRKNV	124	13.7 kDa
CD81	WLRHDPQTTNLLYLELGDKPAPNTFYVGIYIGGGGSGFVNKDQIAKDVKQFYDQALQQAVVDDDANNAKAVVKTFHETLDCCGSSTLTALTTSVLKNNLCPSGSNIISNLFKEDCHQKIDDLFSGKL	127	12.3 kDa

**Table 2:** The codon optimized nucleotide sequence for the synthesis and cloned in PET28a vector with restriction sites

Name	Nucleotide sequence with restriction enzyme site	Accession no
CD9	Nco1:CCATGGGCTGGCTGCGTTTCGACTCTCAGACCAAATCTATCTTCGAGCAAGAAACCAACAACAACAATAGCTCCGGCGGTGGTGGCTCTTATTCTCATAAAGACGAAGTTATTAAAGAAGTGCAGGAATTTTACAAAGACACCTATAACAAACTGAAAACCAAAGACGAGCCGCAGCGCGAAACCCTGAAAGCAATTCACTACGCACTGAACTGTTGCGGCCTGGCAGGTGGTGTGGAGCAGTTCATTTCTGACATCTGCCCGAAAAAAGACGTTCTGGAAACTTTTACCGTTAAGAGCTGCCCGGACGCGATCAAAGAGGTGTTCGATAACAAATTCCACATTCTCGAG:Xho-1	OR166431
CD63	Nco1:CCATGGGCGTTGGTGCACAACTGGTTCTGTCTCAAACCATCATCCAGGGTGCTACCCCAGGTTCCGGTGGTGGTGGTAGCGGTTACGTATTCCGCGACAAAGTCATGTCCGAGTTCAATAACAACTTCCGTCAGCAGATGGAAAACTACCCGAAAAACAACCACACCGCAAGCATTCTGGACCGCATGCAGGCTGACTTCAAATGCTGCGGCGCAGCGAACTACACCGATTGGGAGAAAATTCCGTCCATGAGCAAAAACCGTGTACCGGACAGCTGTTGTATCAACGTTACTGTAGGTTGCGGCATCAACTTTAACGAAAAAGCTATCCACAAAGAAGGTTGCGTGGAAAAAATCGGTGGTTGGCTGCGTAAAAACGTGCTCGAG:Xho-1	OR166432
CD81	Nco1:CCATGGGCTGGCTGCGTCACGATCCTCAGACTACTAACCTGCTGTATCTGGAACTGGGTGATAAACCGGCGCCGAACACCTTTTACGTTGGCATTTACATCGGCGGTGGTGGCTCCGGTTTCGTTAACAAAGACCAGATTGCAAAAGATGTGAAACAGTTCTACGACCAGGCCCTGCAGCAGGCGGTTGTTGACGATGACGCGAACAACGCCAAGGCGGTTGTTAAGACTTTCCACGAGACCCTGGATTGCTGTGGTTCTAGCACCCTGACTGCGCTGACTACCTCTGTTCTGAAAAACAACCTGTGTCCATCTGGTTCTAACATTATCTCTAACCTGTTCAAAGAAGATTGCCACCAGAAAATCGATGACCTGTTTTCCGGTAAACTGCTCGAG:Xho-1	OR166433

### Protein expression and purification

**Fig. 2:**
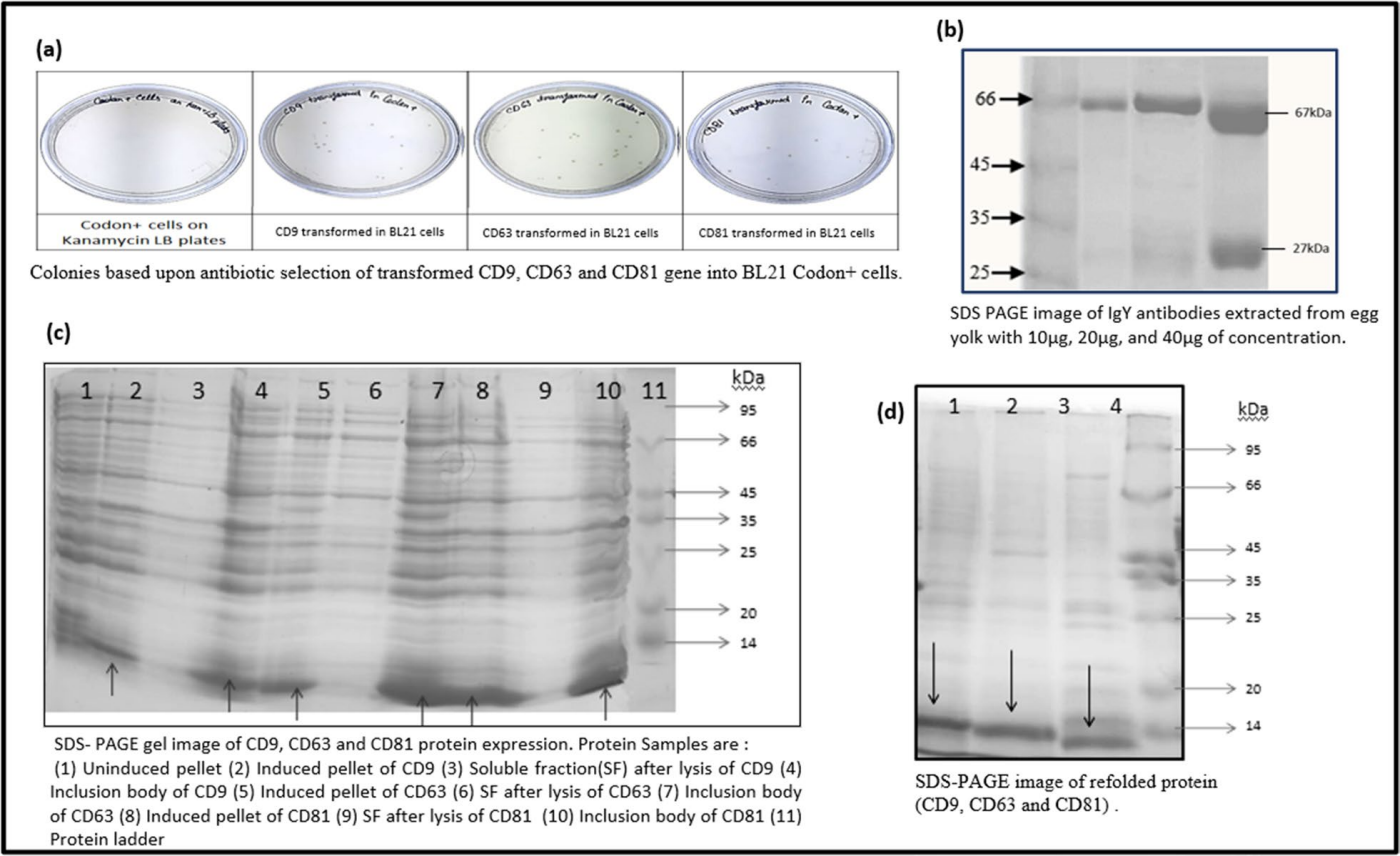
Representing the **a** cloning, **b** antibody purity, **c** identification of desired proteins and **d** purified desired protein

In this investigation, an *E. coli* strain called BL21 was used to express protein for the three tetraspanins, CD9, CD63, and CD81. The acquired colonies grew on an LB medium and were validated using the antibiotic (Kanamycin) selection method (Fig. 2). A single colony has been picked for further expression purposes through the IPTG method. The bacterial pellet was chemically lysed, and the SDS-PAGE result indicated that the desired protein is expressed in the inclusion body (Fig. 2). For CD9, CD63, and CD81, an overexpressed protein band of approximately 12.3 kDa, 13.7 kDa, and 12.3 kDa was observed. The IB was then purified and subjected to additional denaturation and renaturation to acquire its native protein structure. Relying upon the SDS-PAGE image, the purity was estimated to be between 70 and 80% following the final refolded process (Fig. 2).

Along with tetraspanins purification, targeted antibodies are also analyed using SDS PAGE electrophoresis, and the purity of antibodies may slightly vary due to different laboratory conditions or extraction methods. The salt precipitation of antibodies was carried out in two key steps, namely the exclusion of lipids and the precipitation of maximum antibodies, yielding more than 80% purity. SDS-gel confirmed two distinct bands, one of which corresponded to a molecular weight of approximately 68 kDa and the other to approximately 28 kDa (Fig. 2).

### Quantitative analysis of antibodies affinity through ELISA

**Fig. 3:**
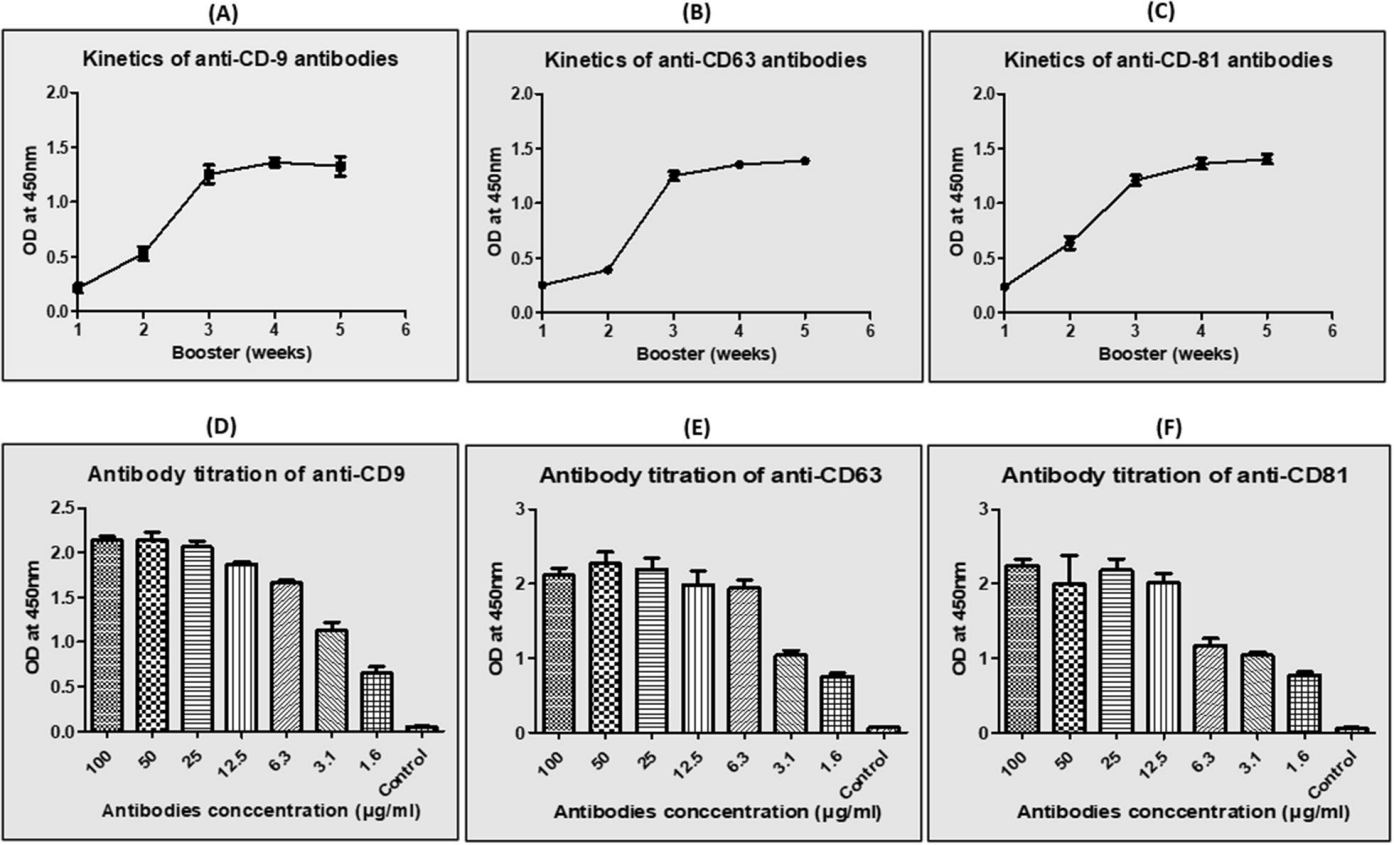
Representing the kinetics of antibodies raising and their titration towards the antigen through ELISA **A** Kinetic of anti CD9 **B** Kinetic of anti-CD63 **C** Kinetic of anti-CD81 **D** Titration of anti CD9 **E** Titration of anti-CD63 **F** Titration of anti-CD81

Anti-tetraspanin antibodies were analysed in two ways: the kinetics of the antibodies in relation to the booster dosage and the titration of the antibodies with the highest affinity and avidity. The kinetics of antibodies were evaluated using an indirect ELISA method to determine the booster dosage at which antibodies acquire their maximum affinity and avidity. This was performed with consistent antigen and antibody concentrations, 0.5 μg/well antigen, and 10 μg/well antibodies. Following that, secondary antibodies (anti-chicken) were added as detecting antibodies at a 1:6000 ratio, and its conjugation is described further below. The three anti-tetraspanin antibodies, CD9, CD63, and CD81, followed the same kinetics studies (Fig. 3A, B and C). The third booster dosage resulted in the highest antibody titer, which increased considerably along with the booster dose. The titer became steady following the third booster dose, indicating that the antibodies had attained their maximal affinity for the antigen. Hence, following the third booster, antibodies were examined for titration to determine which concentration of antibodies is most specific for antigen detection. Indirect-ELISA was used for titration, with a constant concentration of antigen and a variable concentration of primary antibody. All three antibodies acquired a remarkable titer, with the resultant antibody concentration of 100–200 ng capable of detecting around 100 ng of antigen (Fig. 3D, E and F). The titration of all three antibodies was carried out in accordance with the Indirect-ELISA methodology described above.

### Characterization of AuNPs and IgY@AuNPs

**Fig. 4:**
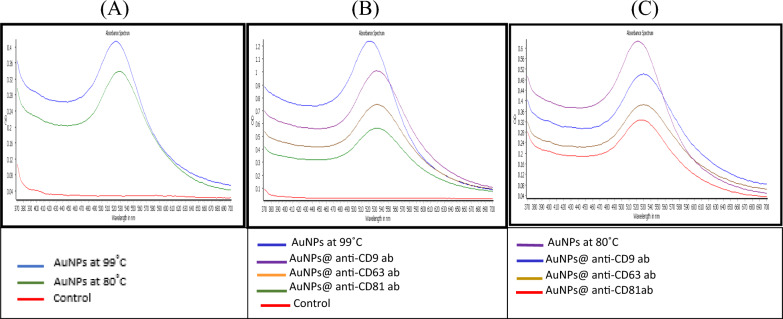
Representing the Wavelength peaks as: **A** UV absorbance spectra of AuNPs prepared at distinct temperature **B** AuNPs prepared at 99 ℃ and conjugated with three different antibodies represented in the graph. **C** AuNPs prepared at 80 ℃and conjugated with three different antibodies represented in the graph

Gold nanoparticles were fabricated at two different temperatures, one at 99 ℃ and the other at 80 ℃, to determine the size and protein binding efficiency of each nanoparticle. The sizes of AuNPs synthesized at 99 ℃ and 80 ℃ determined by TEM are 18 ± 1 and 25 ± 1 nm, respectively, and spherical in shape. However, there was not a major difference between the distinct temperature synthesized nanoparticles indicated above. These nanoparticles were then utilized to bind anti-tetraspanin antibodies, forming a structure known as the IgY@AuNPs complex. Additionally, the UV-spectrophotometer was used to analyse the absorption spectra of AuNPs and IgY@AuNPs complex to determine the maximum absorption wavelength of each. A volume of 200 µl of sample diluted five times in double distilled water was added in flat bottom microtiter plates (Thermo Scientific, USA) for spectroscopic analysis. The AuNPs synthesized at 99 ℃ and 80 ℃ exhibited absorbance peaks at 520 nm and 526 nm, respectively (Fig. 4A). As a negative control, double distilled water was employed in this case. The maximum spectrum of anti-tetraspanin IgY antibodies of CD9, CD63, and CD81 conjugated on AuNPs synthesized at 99 ℃ was observed at approximately 528 nm (Fig. 4B). Although the same three antibodies were coupled with AuNPs synthesized at 80 ℃, the maximum peak obtained for them was about approximately 531 nm (Fig. 4C). The spectrums of all six conjugates (CD9, CD63, and CD81 bound to AuNPs synthesized at 99 ℃ and the same three bound to AuNPs synthesized at 80 ℃) were analysed in contrast to their corresponding nanoparticles.

Furthermore, TEM was used to examine the IgY@AuNPs complex in order to test the isolation of exosomes from serum. Evaluation of the AuNPs-antibody conjugation efficiency was accomplished through the Bradford assay which is well-known assay colorimetric assay to quantify the protein concentration The assay was carried out in accordance with the standard method described in the most recent study, and absorbance was measured at 595 nm (Davatgaran et al. 2018). A concentration of 100 µg of antibodies was introduced to 1 ml of AuNPs, resulting in approximately 50% binding to the nanoparticles. For further exosome isolation from serum, roughly 50 µg of antibodies were bound to 1 ml of AuNPs.

### Exosomes isolation and characterization from serum

**Fig. 5:**
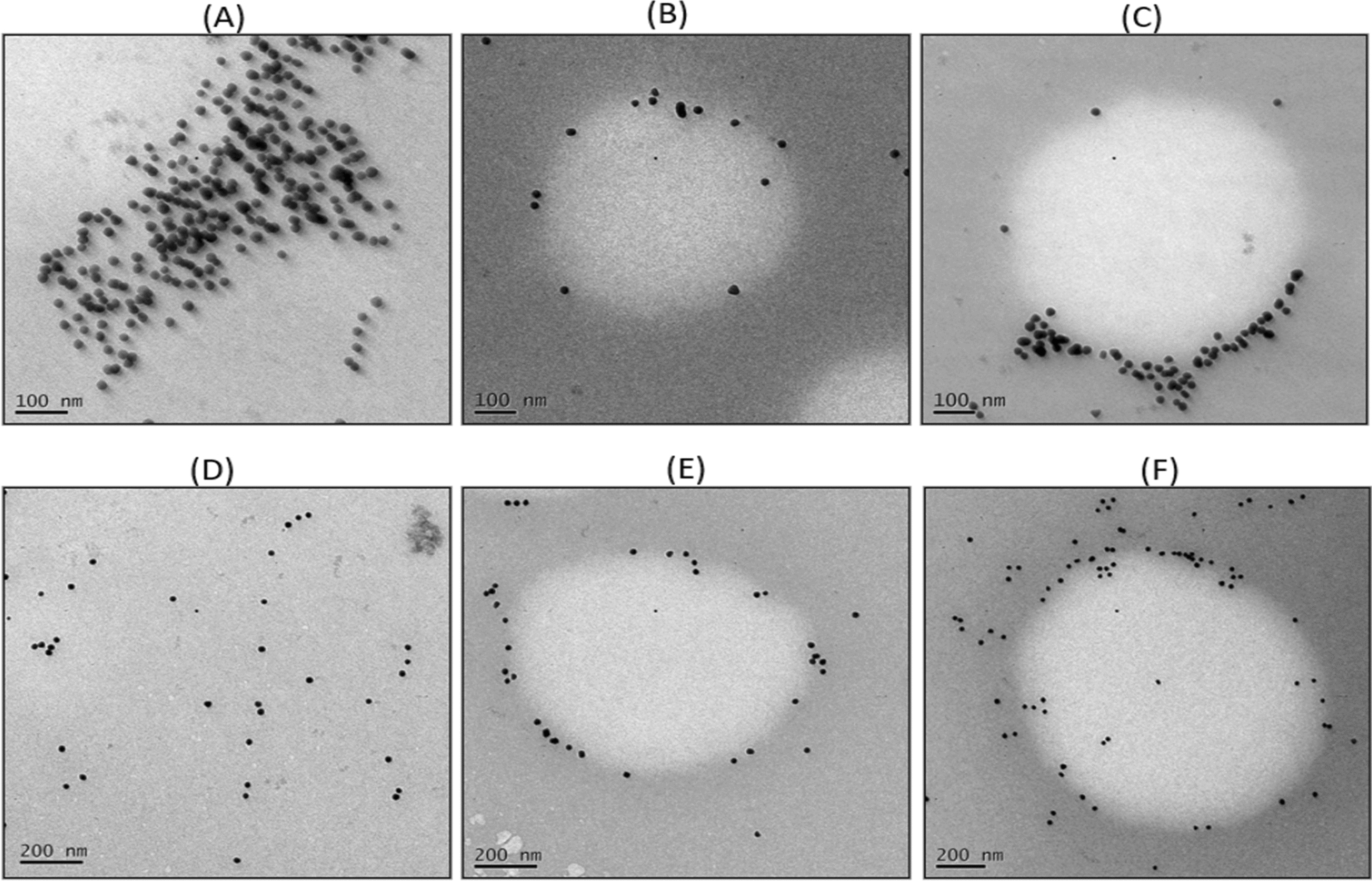
Electron microscopy Data **A** Naked nanoparticles (99 ℃) at a scale of 100 nm results as 18 ± 1 in size **B**, **C** Exosomes captured complex of IgY@AuNPs **D** Naked nanoparticles (80 ℃) at a scale of 200 nm results as a 25 ± 1 nm in size **E**, **F** Exosomes captured complex of IgY@AuNPs

A single antibody conjugation, CD63 antibody conjugated to AuNPs, was employed for the analysis of serum exosome detection. The TEM results indicate that the IgY@AuNPs complex can extract exosomes from human serum (Fig. 5).

Both the AuNPs synthesized at 80 ℃ and 99 ℃ were analysed for exosomes capturing from the serum and TEM image revealed effectively caught exosomes in both cases. The AuNPs fabricated at 99 ℃ were scaled in the TEM at 100 nm, whereas the AuNPs synthesized at 80 ℃ were scaled at 200 nm.

## Discussion

Exosomes have been involved in a variety of fields, including drug delivery and disease diagnosis, yet there is still a shortage of exosome detection tools (Huda et al. 2021; Zhu et al. 2022). Exosomes are naturally cell-secreted signaling molecules with a size range of 20–150 nm comprising a diverse array of biomolecules like as DNA, RNA, proteins, mRNA, microRNA, long noncoding RNA, circular RNA (Zhang et al. 2019; Gurunathan et al. 2021; Gurung et al. 2021). There are several technologies available for isolating exosomes from various biological fluids, including ultracentrifugation, chromatography, and ultrafiltration, as well as a few commercial kits, but each has its own limitations (Pammi et al. 2022). The current work describes a gold nanoparticle (AuNPs) based rapid and successful strategy for exosome detection in human serum utilizing anti-exosomal tetraspanin antibodies. Exosomes, have various surface tetraspanin markers, but CD9, CD63, and CD81 are reported as dominantly expressed and mostly utilized for exosome isolation and purifications (Kowal et al. 2016; Khushman et al. 2017). However, their expression varies depending on the parent cell type, with CD63 being the most addressed as a highly expressed one (Khushman et al. 2017). As a result, three tetraspanins, CD9, CD63, and CD81, have been chosen to raise antibodies for exosome capture. The extracellular region of all three tetraspanin surface markers was adopted for antibody production. Correspondingly, the sequence was codon-optimized (Table.2), cloned in the PET 28a plasmid by Gene Universal, and expressed in the BL21 codon plus strain of *E. coli*. In a study, it was described a variety of expression strains as well as how BL21-Codon Plus (DE3) produced the highest protein yield (Assadi et al. 2008; Robichon et al. 2011).

The extracellular region of CD9, CD63, and CD81 tetraspanin has been retrieved online from NCBI, which provides a wide range of online resources for true biological information and data from 38 distinct databases, including a gene bank, a nucleic acid sequence, a PubMed database, Bio Project, BLAST databases, and so on (Wheeler et al. 2005). Furthermore, the purity of three tetraspanins- exosomes surface marker as well as its targeted antibodies was characterized through SDS-PAGE. The following SDS-PAGE for antibodies revealed 80–90% purity and two distinct bands of roughly 68 kDa and 28 kDa, whereas refolded antigenic extracellular protein revealed 70–80% purity.

Moreover, IgY concentration in egg yolk elevated throughout the immunization span until week 6, when it started to rise rapidly at 2 weeks and stagnated at 4 weeks. As a result, it is possible to conclude that the chicken's immune system takes approximately 2 weeks to give a boost in raising antibodies (Sudjarwo et al. 2017). However, a decrease in titer has been reported in the case of chickens about after 6 weeks of immunization. Even so, a significant decline in titer has been revealed in chickens approximately 6 weeks after immunization (Sudjarwo et al. 2017). Additionally, it was demonstrated that chickens immunized intramuscularly with the antigen, can raise targeted antibodies for more than 200 days, and also provides a high titer and tenfold greater specificity than subcutaneous injection (Sudjarwo et al. 2017). In this article, we demonstrate that a stagnant phase of antibodies titration has arrived after the third immunization as measured by indirect ELISA (Fig. 3). For indirect-ELISA anti- chicken HRP-secondary antibody has been internally conjugated in accordance with a recent article technique and yielded a titer of 1:6000 (Pavliuchenko et al. 2019). Although, along with the antibody kinetics, titration of all three antibodies was optimized using Indirect-ELISA and resulted in a remarkable titer (Fig. 3).

The antibodies were conjugated to AuNPs, which were synthesized following the previously mentioned, traditional Turkevich method for citrate-stabilised gold nanoparticles (Muhammad et al. 2018). Optimization of particles morphology, size, and surface distribution were conducted by Transmission electron microscope and spectrophotometer. As previously reported in several studies, TEM images demonstrate that decreasing the temperature results in a larger diameter of gold nanoparticles (Dong et al. 2020). The nanoparticles were determined spherical in morphology and monodispersed in nature through TEM. The UV–vis absorption spectra of both AuNPs synthesized at 99 ℃ and 80 ℃ show well-defined surface plasmon resonance peaks at 520 nm and 526 nm, respectively (Fig. 4). Furthermore, a shift in the maximum absorbance wavelength of the UV–Vis spectra in contrast to naked AuNPs was interpreted as evidence of antibody conjugation on the AuNPs (Fig. 4). Two different types of nanoparticles, each coupled with three anti-tetraspanins antibodies, were synthesized and characterized here. In comparison to naked nanoparticles, the peak shift was clearly noticed at a difference of 3–4 nm for all six conjugated complexes for each type of AuNP. Hence, this distinct difference in the absorption spectra peaks indicated a successful conjugation and was utilized as a tool for exosome capturing.

In this study PEG was used to stabilize of IgY@AuNPs complex and the stability of IgY@AuNPs was analysed using 0.1 M NaCl solution. Similarly, in a study, Gold nanoparticles introduced to 0.1 M NaCl immediately resulted in a decrease in the absorbance peak, demonstrating their inability to be used in buffer solutions (Christau et al. 2017). Moreover, gold nanoparticles are highly unstable, and various environmental changes, such as pH changes or excessive centrifugation, cause immediate NP aggregation, as evidenced in several studies by the visible colour change (from red to purple) of the AuNPs solution, indicating an increase in particle size (Christau et al. 2017; Polte et al. 2010). Consequently, PEG is considered one of the best polymers for increasing the stability of nanoparticles even after numerous centrifugations during the process (Dallari et al. 2021). Here, the non-covalent immobilization technique was used to conjugate the antibody with nanoparticles, in which antibody was spontaneously absorbed onto the surface of citrate-stabilised nanoparticles with three types of interactions i.e., ionic, hydrophobic, and dative interactions. The attraction between the hydrophobic parts of the antibody and the metal surface results in the formation of a non-covalent bond (Ljungblad et al. 2009). Ionic attraction exists between negatively charged groups on the surface of gold nanoparticles and positively charged groups in antibodies caused by positively charged amino acids and their N-terminal region (Ljungblad et al. 2009; Jazayeri et al. 2016). Furthermore, the dative interactions (coordinate covalent bond) are responsible to form a covalent bond between the AuNPs surface and free sulfhydryl groups of the antibody (Ljungblad et al. 2009; Jazayeri et al. 2016). Before treating with PEG, the antibody and AuNPs complex were stabilized and blocked using BSA due to its nature to prevent the non-specific binding of molecules and so enhance the assay sensitivity. Similarly, in a recent study, BSA was reported to be effective in preventing nanoparticle aggregation (Leopold et al. 2017).

In this article, we provide a more efficient and successful approach for isolating exosomes from human serum, which could potentially be employed for exosome isolation or exosomal cargo exploration. Anti-CD63 antibodies were applied to better understand and characterize the exosomes in human serum. Additionally, the expression of exosomal surface protein receptors was investigated, and observed that CD63 is prevalent in serum, but CD81 appears to be the rarest population (Karimi et al. 2022). However, they came to the conclusion that although CD9-enriched exosomes are also present, they are less numerous than CD63 (Karimi et al. 2022). Additionally, various groups used anti-CD63 antibodies to isolate the population of tiny EVs (Karimi et al. 2022; Pammi et al. 2022). Considering this, the TEM findings demonstrated that both conjugates, which were CD63 conjugated to both the 99 ℃ and 80 ℃ AuNPs, had efficiently captured the exosomes (Fig. 5).

In a nutshell, the primary objective of this study was to establish a rapid, cost-effective, and efficient method of isolating exosomes from biological fluids by simply utilizing immunoaffinity pull-down techniques. This procedure begins with the identification of extracellular sites, then moves on to the production of antigen in bacterial cells, which is then introduced into chickens to produce anti-exosomal antibodies. To extract exosomes from serum, AuNPs were used as an immobilized matrix for antibodies. The novel IgY@AuNPs complex efficiently captures exosomes from bodily fluids in a rapid and cost-effective manner, as determined by TEM. The isolated exosomes could be used for a variety of purposes, including disease diagnosis, drug delivery, and miRNA extraction. Exosomal miRNAs serve as unique molecular markers for monitoring cancer progression and therapy. Early cancer detection could minimise disease-related mortality and increase life expectancy. As exosomal miRNAs considered a circulating diagnostic marker for different types of cancer, are emerging as promising tools for non-invasive screening and targeting of cancer cells in therapy. Several reviews have discussed the significance of serum exosomal miRNAs in a variety of cancers, however using exosomes and their cargo as a diagnostic biomarker still challenging and requires advanced methodologies. Meanwhile, this research could provide a new insight for commercially harvesting exosomes from serum using an immunoaffinity combined with nanotechnology approach. We anticipate that the proposed approach will be valuable in more in-depth research on exosomal subcategories, and it can be applied to different sized small extracellular vesicles.

## Data Availability

The data that support the findings of this study are available on request from the corresponding author. The data are not publicly available due to privacy or ethical restrictions.
